# TUBERCULOSIS IN PEDIATRIC PATIENTS: HOW HAS THE DIAGNOSIS BEEN MADE?

**DOI:** 10.1590/1984-0462/;2017;35;2;00004

**Published:** 2017-05-15

**Authors:** Ana Paula Ghussn Cano, Mariana Tresoldi Neves Romaneli, Ricardo Mendes Pereira, Antonia Teresinha Tresoldi

**Affiliations:** a Faculdade de Ciências Médicas da Universidade Estadual de Campinas (Unicamp), Campinas, SP, Brasil.; b Hospital de Clínicas da Unicamp, Campinas, SP, Brasil.; c Departamento de Pediatria da Faculdade de Ciências Médicas da Unicamp, Campinas, SP, Brasil.

**Keywords:** Pediatrics, tuberculosis, diagnosis

## Abstract

**Objective:**

To describe clinical, radiological, epidemiological, and microbiological characteristics of pediatric patients with diagnosis of tuberculosis in a period of 15 years.

**Methods:**

Retrospective study including children and adolescents younger than 18 years diagnosed with tuberculosis in the Clinical Hospital of the Universidade Estadual de Campinas in São Paulo State, Brazil. Active tuberculosis was defined by the identification of *Mycobacterium tuberculosis* in culture, microscopy, or histopathological examination. Children with positive clinical history and radiological tests who had been exposed to sick adults or with positive tuberculin skin test were also considered as having active tuberculosis. Statistical analysis compared the data obtained from children younger and older than 10 years of age, since they present a disease pattern more similar to adults.

**Results:**

There were 145 identified cases, 61.4% in patients under 10 years of age. The main symptoms reported were coughing (55.9%) and fever (46.9%), and the variables of fever, coughing, weight-loss, and pain were significantly influenced by age, with a higher frequency in older children. Diagnosis was confirmed by culture, microscopy, or histopathology in 67.6% of the cases. The other cases (32.4%) had the diagnosis of tuberculosis based on clinical, radiological, and epidemiological characteristics, as well as tuberculin skin test. The positivity for culture, microscopy, and tuberculin skin test was, respectively, 65.8, 35.7, and 72.3%. History of contact with a sick adult was confirmed in 37.2%, without influence of age.

**Conclusions:**

Diagnosis of tuberculosis in children is still a challenge, since all the confirmation tests have low positivity. These results demonstrate the need for new diagnostic methods and improved strategies for searching sick contacts.

## RESUMO

**Objetivo:**

Descrever o quadro clínico, radiológico e o perfil epidemiológico e microbiológico de pacientes diagnosticados com tuberculose em um período de 15 anos.

**Métodos:**

Estudo retrospectivo de crianças e adolescentes menores de 18 anos diagnosticados com tuberculose no Hospital de Clínicas da Universidade Estadual de Campinas. Tuberculose ativa foi definida pela identificação de *M. tuberculosis* em cultura, baciloscopia ou exame histopatológico ou, ainda, casos com quadro clínico e radiológico compatível e com história de exposição à tuberculose ou com teste tuberculínico positivo. Foi realizada análise estatística, com comparação entre os dados de crianças menores e maiores de 10 anos, já que estas apresentam doença com padrão mais próximo do adulto.

**Resultados:**

Foram identificados 145 casos, 61,4% em pacientes menores de 10 anos. Os principais sintomas relatados foram tosse (55,9%) e febre (46,9%), sendo que as variáveis febre, tosse, emagrecimento e dor se associaram à idade, estando mais presentes em crianças maiores de 10 anos. O diagnóstico foi confirmado (cultura, baciloscopia ou histopatológico) em 67,6% dos casos. Os demais (32,4%) tiveram seu diagnóstico realizado com base no quadro clínico, radiológico, epidemiológico e teste tuberculínico. A positividade da cultura, da baciloscopia e do teste tuberculínico foram, respectivamente, 65,8; 35,7 e 72,3%. História de contato com adulto tuberculoso foi confirmada em 37,2% dos casos.

**Conclusões:**

O diagnóstico de tuberculose em pediatria é complexo, considerando-se que todos os parâmetros usados têm baixa positividade. Os resultados apontam a necessidade de novos métodos diagnósticos e também de melhorias nas estratégias de busca de comunicantes.

## INTRODUCTION

TB was defined by the detection of *Mycobacterium tuberculosis* in culture, smear microscopy (bacillus resistant acid - BAAR), or histopathology indicative of TB, evidencing a chronic granulomatous inflammatory process with caseous necrosis. Children with a compatible clinical and radiological condition and a history of exposure to TB or positive tuberculin test were also considered cases of active TB (≥5 mm in non-BCG vaccinated children, children vaccinated for more than 2 years and immunosuppressed, or ≥10 mm in children vaccinated for less Tuberculosis (TB) in Brazil presents a major public health challenge. Despite current efforts to control it, data from the World Health Organization (WHO) indicate that Brazil is among 22 countries with the highest number of TB cases, accounting for 82% of global cases and 75% of cases involving children.^1^ There are approximately 1 million TB cases in the world’s child population, and the disease accounts for 130,000 deaths per year, making TB one of the 10 leading causes of death among children worldwide.^2^ According to the SINAN reporting system, 83,617 TB cases were registered in Brazil in 2015, of which 7,106 cases (8.5%) occurred in children under 19 years of age.^3^

The major challenge related to childhood TB is its diagnosis, since there is no examination to date that can be considered a gold standard.^4^ ^,^ ^5^ Diagnostic techniques classically used in adults have low sensitivity and specificity in children and confirmation by bacteriological identification is not always possible. Thus, treatment often begins in the absence of isolation of the mycobacteria and is based only on the following three factors:

1. clinical and radiological findings;
2. positive tuberculin skin test; and
3. contact with an adult with TB.^6^ ^,^ ^7^

However, even clinical and radiological findings of TB in children are difficult to analyze. While adolescents express a disease with a pattern similar to that of adults, younger children often present a nonspecific and oligosymptomatic presentation, therefore constituting the age group of greater diagnostic difficulty and higher risk of progression to severe illness and death.^6^ ^,^ ^8^

Although the diagnostic difficulties in the pediatric age group are many, few studies have been conducted to evaluate how the diagnosis of childhood TB is carried out in Brazil.^9^ ^,^ ^10^ ^,^ ^11^ Such a scarcity of studies impairs the identification and monitoring of cases and hinders the development of public policies aimed at the prevention and control of the disease.

This study aims to describe the clinical and radiological profile as well as the epidemiological and microbiological profile of children and adolescents diagnosed with TB at any time between 1999 and 2014 at the Hospital of the State University of Campinas (HC-Unicamp).

## METHOD

A retrospective study was carried out and approved by the Research Ethics Committee of Unicamp under opinion number 570,646. The data consisted of records of children and adolescents up to 18 years of age who were diagnosed with TB at HC Unicamp sometime during the 15-year period between 1999 and 2014. Active TB was defined by the detection of *Mycobacterium tuberculosis* in culture, smear microscopy (bacillus resistant acid - BAAR), or histopathology indicative of TB, evidencing a chronic granulomatous inflammatory process with caseous necrosis. Children with a compatible clinical and radiological condition and a history of exposure to TB or positive tuberculin test were also considered cases of active TB (≥5 mm in non-BCG vaccinated, vaccinated for more than 2 years and immunosuppressed, or ≥ 10 mm in vaccinated for less than 2 years). Patients who did not fit these definitions were excluded even if treatment was prescribed. In addition, patients who began follow-up in the post-relapse or retreatment service were likewise excluded, as were cases of latent TB and BCGitis infection (infection caused by the BCG vaccine at the site of application and in regional lymph nodes). The Epidemiological Surveillance Nucleus (NVE) of the Surveillance System at HC Unicamp (TB Web) identified the cases from 2007 to 2014, and the Patient Information System of HC Unicamp identified the cases from 1999 to 2006. Cases were identified according to the International Classification of Diseases (ICD).

The information collected from the medical records included personal identification data, clinical history, physical examination, form of presentation of the disease, radiological and microbiological studies, epidemiological data, and the conduct and outcome of each case. Whenever available, descriptions of images of radiological findings in the medical records or official reports were also taken into consideration.

Statistical analysis was performed with descriptions of variables according to their frequencies, and comparison between them used the chi-square test or Fisher’s exact test. The comparison between the data of children was made by age group, as those older than 10 years present a disease pattern similar to that of adults, whereas the younger age group presents particular and unique manifestations, different clinical conditions, and distinct diagnostic difficulties.^8^

## RESULTS

We identified 145 individuals with active TB disease: 69 were males and 76 were females. The median age was 7 years. A total of 89 children (61.4%) were younger than 10 years of age and 56 (38.6%) were older than 10 years. Approximately 30% of the children started follow-up at the health service with up to 4 weeks of clinical history; 15% from 5 to 8 weeks; and 15%, from 9 to 16 weeks. There is no information on the time of clinical history in 25% of the cases, and the remaining 15% varied with durations of clinical history ranging from extremes of 6 months to 14 years.

Among the types of active disease, 88 cases (60.7%) had pulmonary type and 57 (39.3%) had extrapulmonary types. The age group did not significantly influence the presentation of the disease as pulmonary or extrapulmonary (*p*=0.631). However, there were more cases of pleural TB in patients older than 10 years and all cases of bone TB occurred uniquely in patients younger than 10 years of age (Table 1).

**Table 1:**
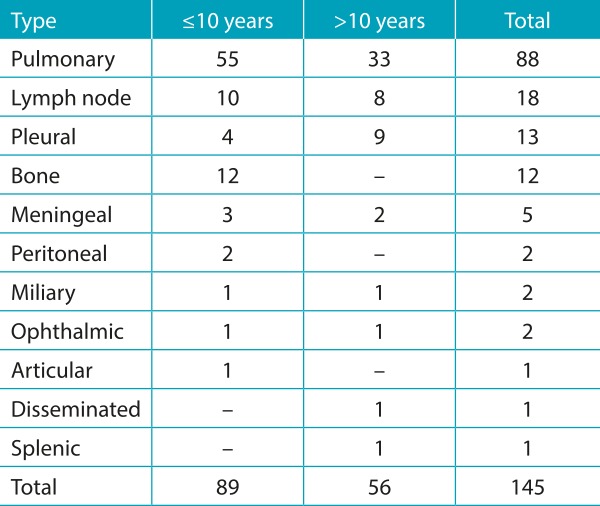
Distribution of tuberculosis presentation by age group.

The main clinical history data are described in Table 2. Coughing, fever, and weight loss were the most commonly reported symptoms. The variables of fever, coughing, weight loss, and pain were significantly influenced by age, with a higher frequency in children older than 10 years of age. The pain variable was present in 13 patients with pulmonary type (chest pain - ventilatory dependent); in 9 patients with the pleural type (pleuritic pain); and in 4 patients with the bone type (bone pain with claudication). The other cases were described in patients with the meningeal type (2 cases - headache), lymph node type (2 cases - pain in the affected lymph nodes), articular type (1 case - coxofemoral pain), ophthalmic type (1 case - ocular pain), and miliary type (1 case - chest pain).

**Table 2:**
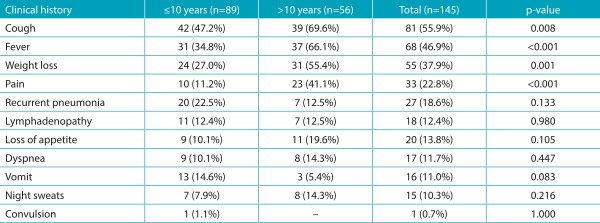
Distribution of signs and symptoms of tuberculosis according to the age group.

With respect to the physical examination, the main data were altered pulmonary auscultation (53 cases/36.6%), lymphadenopathy (45 cases; 31.0%), fever on admission (25 cases; 17.2%), and malnutrition (11 cases; 7.6%).

Of the 145 cases examined in the study, 98 (67.6%) had a diagnosis of TB confirmed by culture, bacilloscopy (BAAR survey), or a histopathology indicative of TB, evidencing chronic granulomatous inflammatory process with caseous necrosis. In the majority of the cases (63 of 98 cases, or 64.3%), confirmation was made with a positive result in only one of these three confirmatory tests. In 33 cases (33.7%), confirmation was made with a positive result in two of these tests, and in 2 cases (2.0%) there was a positive result in all three tests. There were more confirmatory examinations for the extrapulmonary conditions (*p*<0.001). Diagnostic confirmation correlated with age (*p*=0.025), with 60.7% diagnostic confirmation among those under 10 years of age, and 78.6% among patients older than 10 years. The remaining cases (47 cases; 32.4%) were diagnosed based on the clinical condition, radiological aspects, tuberculin skin test, and history of contact with adult tuberculosis without bacteriological confirmation. Positive results in the culture, bacilloscopy, and tuberculin skin test are presented in Table 3. Tuberculin skin test has turned positive in 12 patients (8.3% of the total, representing 20% of those with a positive tuberculin skin test).

**Table 3:**
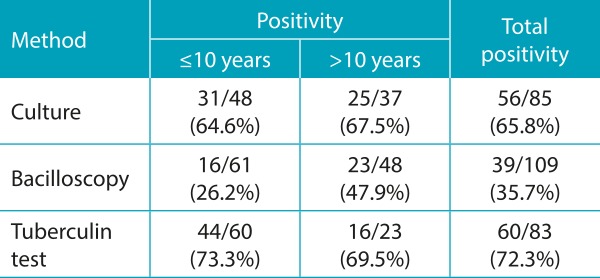
Positivity of culture, bacilloscopy, and tuberculin skin test according to age group (positive results / total of examinations performed).

Culture was performed in 85 cases, with mycobacteria identified in 56 cases (65.8%), of which 44 cases were pulmonary TB and 12 cases were extra-pulmonary TB. This corresponds with 50% and 21% of pulmonary and extrapulmonary types, respectively. In the pulmonary type, culture was positive in the following materials: sputum (25 cases), gastric lavage (11 cases), bronchoalveolar lavage (5 cases), tracheal secretion (2 cases), and lung biopsy (1 case). Gastric lavage analysis was performed in 20 patients, of which smear microscopy was positive in 5 patients and culture was positive in 11 patients.

The histopathological examination confirmed the diagnosis of TB in 41 cases. The main sites from which biopsies were extracted were lymph node (23 cases), bone (7 cases), and pleura (6 cases), as well as other less frequent sites such as liver, spleen, sclera, central nervous system, and synovium (1 case each). In addition to the ganglion form, lymph node biopsy contributed to the diagnosis in three cases of pulmonary TB, one case of miliary TB, and one case of peritoneal TB.

Radiological alterations were present in 94 (81%) of 116 cases in which the examination was performed. The main alterations were the presence of opacities in the pulmonary parenchyma (33 cases; 22.7%), hilar adenopathy (13 cases; 9.0%), pleural effusion (13 cases; 9.0%), cavitation (8 cases; 5.5%), and miliary pattern (2 cases; 1.4%). Computed tomography was performed in 39 cases (26.9%), with evidence of alteration in 34 (87.2%), of which 24 (70.6%) were cases of pulmonary TB and the remaining cases were extrapulmonary.

In 14 cases (9.7%) diagnosed with TB, there was a previous diagnosis of HIV infection. Investigation of co-infection was carried out in 73 other cases, but it was negative in all the cases. Contact with adult tuberculosis was confirmed in 54 cases (37.2%), with no relation to age (*p*=0.972). There was no information on the search for contacts in 59 cases (40.7%). Previous vaccination with BCG was reported in 89 cases (61.4%), being absent in only 2 cases (1.4%) and without information in 54 cases (37.2%). Of the 2 cases of miliary TB, 1 child had been vaccinated and there was no information about the other. In the meningeal type, 4 children had been vaccinated and there was no information about 1 of the patients.

Of the patients considered, all started adequate treatment and 120 (82.8%) patients were cured; 12 (8.3%) patients performed follow-up in the basic health network, and there were no records of the outcome of these cases; 5 (3.4%) patients abandoned treatment; and 4 (2.8%) patients presented recurrence of the disease. One case (0.7%) was still receiving treatment at the time of data collection; 2 (1.4%) resulted in death (one case of miliary TB and one case of pulmonary TB with HIV co-infection), and there is no information on the follow-up of 1 patient. Drug hepatitis was diagnosed in 6 (4.1%) patients.

## DISCUSSION

The results demonstrate the complexity of the diagnosis of TB in the pediatric age group, as well as its complexity in the absence of bacteriological confirmation. Difficulty in diagnosis among children is evident because of its delay: despite the lack of information about the duration of clinical history in a quarter of the cases, it is notable that only 30% of children started treatment within the first four weeks of clinical history, meaning that many others endured months and even years of clinical history before obtaining a diagnosis.

The most commonly reported symptoms were coughing (55.9%), fever (46.9%), and weight loss (37.9%), which were also described as the main symptoms in similar cohorts.^12^ ^,^ ^13^ ^,^ ^14^ The occurrence of these symptoms were correlated with age: they were more present in the group of children over 10 years of age. The low frequency of these symptoms in the patients younger than 10 years of age and the oligosymptomatic nature of TB in this age group reinforce the difficulty of diagnosing the disease in younger children.

The literature demonstrates that although mycobacteria research and culture are the most commonly used diagnostic methods when TB is suspected, less than 20% of the children with the diagnosis have positive smear microscopy and the culture detects *M. tuberculosis* in less than 50% of the cases.^6^ ^,^ ^7^ The results of this current study are similar, with smear microscopy positive in 35.7% of cases and positive culture detected in 65.8% cases. These data are consistent with the literature, which rarely presents bacteriological confirmation in children and confirms the greatest diagnostic difficulty in children younger than 5 years of age.^2^ ^,^ ^15^ ^,^ ^16^

The low positivity of bacteriological examinations in the pediatric age group can be explained by the fact that TB in children is usually paucibacillary. This is due to the fact that most of the time these children are unable to spit voluntarily, a challenge that can compromise the diagnosis in cases of pulmonary TB. The alternative method to obtain sputum in these cases is the collection of gastric lavage, a relatively invasive method that requires hospitalization for three days. In this study, gastric lavage culture confirmed the presence of mycobacteria in 55% of the cases in which it was investigated.

In the absence of bacteriological confirmation and other methods in the public service system to confirm the diagnosis of TB, treatment is prescribed using the three factors previously mentioned (clinical radiological criteria, tuberculin skin test, and epidemiology). These criteria are analyzed using a point system proposed by the Ministry of Health in 2010 and validated in our country for children not infected with HIV. This system considers the nutritional status of the child and assigns a score that classifies the diagnosis of pulmonary TB as “unlikely” (≤25 points), “possible” (between 30 and 35 points) and “very likely” (≥40 points).^15^ ^,^ ^17^ Studies that validated the use of this score in Brazil determine that the intermediate punctuation limits for indication of treatment (“possible” diagnosis) increase the sensitivity of the algorithm from 58% to 89%, but also lead to a drop in specificity from 98% to 86%.^15^ Consequently, an increase in false positives may occur, which means that approximately 30% of patients starting treatment for TB might be carriers of other diseases.^18^ The analysis of the use of the point system was not possible in this study due to the long time span considered (1999-2014), as it includes periods prior to the implementation of the system by the Ministry of Health.

More recently developed methods, such as polymerase chain reaction techniques, microscopic observation drug susceptibility (MODS), bacteriophage amplification, and assays for the detection of interferon-gamma (IFN ) (Interferon-Gamma Release Assays - IGRAS) have not yet been validated for use in children or are not yet available for use in Brazilian public services and have not been used for diagnosis in any of the cases reported in this study.^17^ Research on IGRAS has received particular attention today and includes methods such as QuantiFERON TB Gold or T SPOT.*TB*. Recent studies evaluating these methods and comparing their performance with the tuberculin test have indicated that the IGRAS method achieves superior specificity compared with the tuberculin test. This supports the possibility of its use in place of the tuberculin test and as a form of effective disease screening in children.^19^ ^,^ ^20^ ^,^ ^21^ Nevertheless, the new diagnostic techniques require further studies involving the pediatric age group in order to define parameters that guide their use and to evaluate the cost effectiveness of their use in the public health system.^5^

As children can only be infected after birth by close contact with a bacillary TB carrier, confirmation of the disease in the pediatric age group is a sentinel event that alerts to the presence of adults with tuberculosis in the child’s life.^16^ In this study, there was a sick adult in 37.2% of the cases. Moreover, 50% of TB cases occurred in children under 7 years of age, the age range in which the child’s main contacts are in the home environment.

This fact evidences the need to actively seek out adult patients at home whenever a child with TB is identified. Failure to detect the adult source of the disease is a risk to the child’s own treatment (as the child remains susceptible to re-infection), and is a greater public health issue as it does not prevent the transmission of the disease to other adults. Thus, there is a need for greater investment in strategies for epidemiological surveillance of TB with the implementation of a systematized method of searching for contagious contacts.^22^ ^,^ ^23^ ^,^ ^24^

Regarding HIV TB co-infection, this occurred in 9.7% of the cases. All of these patients were previously diagnosed with HIV and received follow-up care at HC Unicamp. The high percentage is due to the fact that Unicamp is a reference center for HIV, keeping track of infected children sent from the interior of the state of São Paulo and all of Brazil. However, the need to investigate HIV in patients diagnosed with TB (even in the pediatric age group) is more urgent and imperative given that TB is found to be one of the main causes of death in HIV-positive patients. Early identification of these cases may lead to a more effective treatment and better control of the underlying disease.^17^

Considering that this was a retrospective study, the researchers could only rely on the information available in the patients’ charts. In some cases, this information was incomplete or parts were missing, therefore impairing some conclusions. It is worth mentioning that the authors believe that the 100% positivity of histopathological examinations can be explained by the method applied in the research, that is to say, from the identification of CIDs related to TB. Indeed, when the examination was inconclusive or identified another agent, the CID did not detect such a case in the database search.

In summary, the diagnosis of TB in children remains challenging. The results of this study echo the findings of pre-existing literature and illustrate both the diagnostic difficulty in children and the health system’s need for new, cost-effective diagnostic methods that have been validated for the pediatric population. Furthermore, strategies aimed at identifying contagious carriers should be reviewed in order to ensure a break in the transmission link of the disease. When coupled together, these actions may provide better control of TB in Brazil.
